# Characterising the Yellow Muscle Phenotype of Common Carp by Muscle Carotenoids, Fatty Acid Composition and Multi-Omics Analysis

**DOI:** 10.3390/ani16213331

**Published:** 2026-10-22

**Authors:** Shunke Zhang, Tingyu Zhang, Pengcheng Duan, Jianpeng Zhang, Ying Pan, Chuanyu Li, Bojun Chen, Zhiyang Li, Baoyue Lu

**Affiliations:** Key Laboratory of Aquatic Healthy Breeding and Nutrition Regulation of Guangxi Universities, College of Animal Science and Technology, Guangxi University, Nanning 530004, China; sk18260965227@163.com (S.Z.); 15369917819@163.com (T.Z.); 15069212276@163.com (P.D.); jpzhang@gxu.edu.cn (J.Z.); yingpan@gxu.edu.cn (Y.P.); cy17663219901@163.com (C.L.); bojun319@163.com (B.C.); l2959619253@outlook.com (Z.L.)

**Keywords:** carotenoids, muscle color, lipid metabolism, multi-omics analysis, *Cyprinus carpio*

## Abstract

Muscle color affects the market value of common carp (*Cyprinus carpio*), but the pigmentary and metabolic basis of the yellow muscle phenotype remains unclear. In this study, we compared muscle carotenoids, fatty acid profiles, and multi-omics data between wild yellow muscle (YC) and normal muscle (NC) common carp. YC showed significantly higher yellowness (b*) value, but there were no significant differences in the lightness (L*) and redness (a*) values. Total carotenoid was higher in YC and positively correlated with b* value. YC had higher saturated fatty acids (SFA) and n-3 polyunsaturated fatty acids (n-3 PUFA) proportions, including eicosapentaenoic acid (EPA) and docosahexaenoic acid (DHA), but lower n-6 and total PUFA, despite unchanged crude fat content. Total carotenoid content correlated with multiple fatty acids but not crude fat. Multi-omics analyses converged on glycerophospholipid metabolism, unsaturated fatty acid biosynthesis, and arachidonic acid metabolism, and *cd36*, *scarb1*, and *bco2* were upregulated in YC muscle. In summary, these findings indicate that carotenoid accumulation underlies enhanced yellowness, while lipid remodeling and related transport processes may facilitate carotenoid retention in YC.

## Simple Summary

Yellow muscle has been observed naturally in wild populations of common carp, but the biological basis of this trait remains unclear. In this study, we compared common carp with yellow muscle and those showing normal white muscle. Yellow muscle contained more carotenoid pigments and showed clear differences in fatty acid composition, whereas total fat content was similar between the two groups. These findings indicate that carotenoid accumulation and fatty acid composition are closely associated with the yellow muscle trait. These results also provide a basis for understanding muscle color formation and for the future breeding of stable yellow muscle common carp.

## 1. Introduction

The color of fish muscle is an important determinant of sensory quality and market value, with bright colors such as red and yellow generally being more attractive to consumers and commanding higher market prices [1]. Muscle color is influenced by multiple factors, including pigment composition, tissue structure, and biochemical status. Among these, carotenoids can impart yellow to red coloration to tissues, and their composition and deposition levels constitute an important pigmentary basis for muscle color variation in many fish species. In commercially important salmonids, carotenoid deposition in muscle is widely used as an important criterion for quality grading and market pricing [2]. Carotenoids in fish originate from dietary intake. They are absorbed in the intestine and enter the body via the bloodstream. Their final accumulation levels in tissues are determined by a combination of tissue uptake, metabolic conversion, and degradation [3,4]. Although these processes have been relatively well studied in salmonids, carotenoid utilization efficiency, tissue distribution, and associated molecular responses vary markedly among different fish species and even among tissues within the same individual [5,6].

Common carp is an important aquaculture species in China, and research on its muscle quality has primarily focused on nutritional composition, lipid deposition, and flavor components [7,8,9]. Regarding color-related traits, most studies have focused on pigmentation of the skin and scales or evaluated the effects of dietary carotenoid supplementation on body and muscle coloration [10,11,12]. To date, naturally occurring yellow muscle common carp in wild populations have not received sufficient attention, and their characteristics have not been fully described. Such individuals provide unique biological material for investigating muscle coloration, pigment accumulation, and the associated metabolic and molecular changes in fish. However, the major pigments responsible for this coloration, lipid composition, and the underlying molecular mechanisms of the yellow muscle phenotype remain unclear.

Numerous studies have shown that carotenoids are highly lipophilic, and their absorption, transport, and tissue distribution are closely associated with lipid and lipoprotein systems [13,14,15,16,17]. In addition to total muscle lipid content, fatty acid composition, membrane lipid components such as glycerophospholipids, and lipoprotein-mediated transport may also affect the local lipid environment of muscle and the distribution and retention of lipophilic molecules [18,19]. Studies in several fish species have indicated that muscle lipid characteristics are associated with carotenoid content and coloration. For example, in Atlantic salmon (*Salmo salar*), individuals exhibiting different muscle color phenotypes showed differences in both fatty acid composition and carotenoid content [20]. In Arctic char (*Salvelinus alpinus*), dietary lipid levels have also been associated with carotenoid deposition and muscle colorimetric parameters [21]. Similarly in a comparison between wild-caught and pond-cultured Yellow River carp (*Cyprinus carpio* haematopterus), changes in fatty acid composition coincided with differences in carotenoid metabolite profiles [11]. Collectively, these findings suggest that carotenoid accumulation in muscle may be closely associated with lipid availability, fatty acid composition, and the associated lipid metabolic state. Therefore, further research is needed to determine whether carotenoids are the primary pigmentary basis for the yellow muscle phenotype in common carp, and their accumulation in muscles in relation to crude fat content, fatty acid composition, lipid metabolism, and transport, remains to be studied.

In this study, we analyzed muscle color parameters, total carotenoid content, crude fat content, and fatty acid composition between yellow muscle common carp (YC) and normal muscle common carp (NC). By combining these analyses with untargeted metabolomics, transcriptomics, data-independent acquisition (DIA)-based quantitative proteomics, and qRT-PCR, we elucidated the differences in muscle color phenotypes between the YC and NC group in terms of pigment accumulation, lipid composition, and related molecular characteristics. Furthermore, we identified the chromatic characteristics of yellow muscle and their relationship with carotenoid accumulation, and screened candidate pathways and molecules associated with carotenoid accumulation, lipid metabolism, and the transport of lipophilic compounds, thereby laying the foundation for the breeding of a stable yellow muscle common carp strain.

## 2. Materials and Methods

### 2.1. Animals and Sample Collection

**Figure 1:**
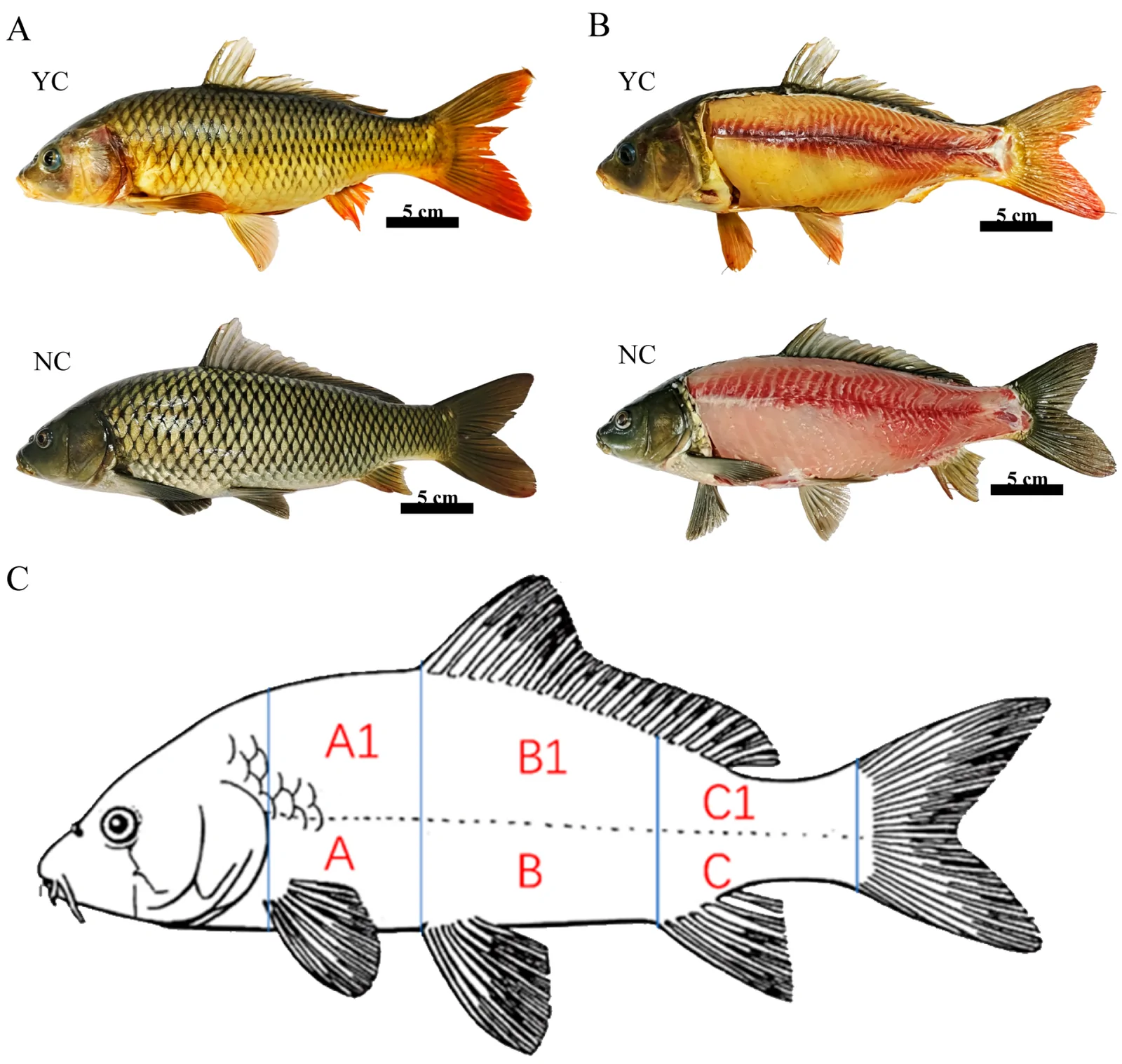
Representative phenotypes of Yellow muscle common carp (YC) and Normal muscle common carp (NC), muscle sampling region, and muscle color measurement sites: (**A**) External appearance of YC and NC; (**B**) muscle appearance of YC and NC; (**C**) schematic illustration of the muscle color measurement sites on the lateral side of common carp. The dashed line indicates the lateral line. Three sampling transects (A, B, and C) were established perpendicular to the lateral line, located between the pectoral and pelvic fins, between the pelvic and anal fins, and between the anal fin and caudal peduncle, respectively. Two measurement sites were designated within each transect, one on the dorsal side (A1, B1, and C1) and one on the ventral side (A, B, and C), giving a total of six measurement sites.

All fish care and handling procedures were approved by the Animal Ethics Committee of Guangxi University (approval no. GXU-2026-302). Both YC and NC were captured on the same day during the same fishing operation in the Yongjiang River basin in Nanning, Guangxi, China. Common carp individuals with a conspicuous yellow coloration on the ventral side between the pectoral and anal fins were preliminarily identified as YC candidate individuals, while fish lacking this characteristic yellow coloration were considered NC individuals. After capture, all experimental fish were reared under standardized laboratory conditions for 15 days to eliminate the effects of stress and physiological disturbances caused by capture and transport on the experimental results. All experimental fish were reared in a 1.5 × 1.5 × 6.0 m tank, with dissolved oxygen maintained at 5–6 mg/L. Approximately one-fifth of the rearing water was replaced every 2 days, and the water temperature was maintained at 25–27 °C. The fish were fed once daily with “Tongwei 180” commercial feed (Tongwei Agricultural Development Co., Ltd., Tianjin, China), which contained no added carotenoids, at a daily feeding rate of approximately 2% of body weight. Since all experimental fish were caught from the same river area in the wild, their feeding history prior to capture was unknown, however, it is reasonable to assume that all experimental fish had access to the same food source in the wild. In addition, since these experimental fish were caught in the wild, their exact ages are unknown, however, all experimental fish had reached sexual maturity. The YC group consisted of 4 males and 3 females, while the NC group consisted of 5 males and 2 females. All experimental fish were fasted for 24 h before tissue sample collection. After dissection, the final classification of YC and NC was based on the gross muscle color phenotype, while CIE 1976 Lab* color space (CIELAB) measurements were subsequently used to quantitatively characterize the predefined muscle color phenotypes. No significant differences in body length (31.21 ± 2.25 cm vs. 29.41 ± 3.12 cm, *p* > 0.05) or body weight (693.15 ± 62.68 g vs. 644.37 ± 19.51 g, *p* > 0.05) were observed between the YC and NC groups. Representative individuals and their muscle phenotypes are shown in Figure 1A,B.

Fish were anesthetized with eugenol (Hushi, CAS No. 97-53-0) before sampling. After removing the skin and red muscle, the white muscle located on the lateral side of the trunk between the pelvic and anal fins and below the lateral line was exposed. Muscle color measurements were then taken immediately, followed by the collection of muscle samples (Figure 1C). Sampling of each fish was completed within 5 min to minimize potential effects of environmental exposure and oxidation on the molecular profiles of the samples. Approximately 10 g of muscle tissue was collected from the same anatomical region of each individual, divided into several equal portions, rapidly frozen in liquid nitrogen, and stored at −80 °C for metabolomics, transcriptomics, and proteomics analyses. Seven biological replicates in each group were used for each omics analysis (*n* = 7).

### 2.2. Muscle Color Measurement

Muscle color parameters were measured using a portable colorimeter SC-100 (Shenzhen ThreeNH Technology Co., Ltd., Shenzhen, China), which was automatically calibrated before measurement. After removal of the skin and subcutaneous tissues, six fixed measurement sites (A, B, C, A1, B1, and C1) were selected on the lateral surface of each fish. Each point was measured three consecutive times, and the mean value was used as the color measurement for that point, with a coefficient of variation (CV) < 5%. Color parameters were recorded according to the CIELAB color space: L* represents lightness, a* redness, and b* yellowness [22,23]. All measurements were performed by the same operator under identical experimental conditions to minimize variability caused by operator and environmental factors. To analyze the correlation between total carotenoid content and muscle yellowness, the b* values measured at site B and the total carotenoid content measured in the muscle of the site B region were used. The b* value for each fish was calculated as the average of three consecutive measurements taken at site B.

### 2.3. Determination of Total Carotenoid Content in Muscle

Total carotenoid content in muscle was determined using a modified organic solvent extraction method [24]. Under dark conditions, 0.5 g of frozen muscle tissue was thoroughly homogenized with pre-chilled acetone. After shaking extraction, the samples were centrifuged and the supernatant was collected. The remaining tissue residue was repeatedly extracted with fresh acetone until it became nearly colorless. The acetone extracts were combined and subjected to liquid–liquid partitioning with a mixture of n-hexane and anhydrous diethyl ether (1:1, *v*/*v*), followed by the addition of an appropriate volume of distilled water to facilitate phase separation. After phase separation, the upper organic phase was collected, and the extraction was repeated once. Subsequently, all organic phases were combined and brought to a fixed volume.

Using the mixture of n-hexane and anhydrous diethyl ether as the blank, the absorbance was measured at a wavelength of 450 nm using a microplate reader. A standard curve was prepared using β-carotene, and total carotenoid content was calculated based on the standard curve. Results were expressed as β-carotene equivalents based on wet weight (mg g^−1^ WW). All procedures were performed under light-protected conditions to minimize photodegradation of carotenoids.

### 2.4. Untargeted Metabolomics Analusis

Untargeted metabolomics analysis of muscle samples was performed by a third-party service provider (Majorbio Bio-Pharm Technology Co., Ltd., Shanghai, China). Sample extraction, LC–MS/MS analysis, and data processing were conducted with reference to a previously published untargeted metabolomics study of aquatic animal tissues, with parameters adapted to the present study [25]. Seven muscle samples from each group were used for analysis. Approximately 100 mg of frozen muscle tissue was placed in a 2 mL centrifuge tube with a 6 mm grinding bead and extracted with 800 μL of methanol/water (4:1, *v*/*v*) containing four internal standards, including L-2-chlorophenylalanine (0.02 mg/mL). Samples were ground for 6 min at −10 °C and 50 Hz, followed by ultrasonic extraction for 30 min at 5 °C and 40 kHz. The samples were then incubated at −20 °C for 30 min and centrifuged at 13,000× *g* for 15 min at 4 °C. The supernatants were transferred to sample vials for LC–MS/MS analysis. A pooled quality control (QC) sample was prepared by mixing equal volumes of all sample extracts and was analyzed once every 5–10 samples to monitor analytical reproducibility.

Metabolite profiling was performed using a UHPLC-Orbitrap Exploris 240 system (Thermo Fisher Scientific, Bremen, Germany) equipped with an ACQUITY HSS T3 column (100 mm × 2.1 mm i.d., 1.8 μm; Waters Corporation, Milford, MA, USA). The mobile phase consisted of 0.1% formic acid in water/acetonitrile (95:5, *v*/*v*) as solvent A and 0.1% formic acid in acetonitrile/isopropanol/water (47.5:47.5:5, *v*/*v*/*v*) as solvent B. The gradient program (solvent B) was: positive mode, 0–3.0 min, 0–20%; 3.0–4.5 min, 20–35%; 4.5–5.0 min, 35–100%; 5.0–6.3 min, 100%; 6.3–6.4 min, 100–0%; 6.4–8.0 min, 0%; negative mode, 0–1.5 min, 0–5%; 1.5–2.0 min, 5–10%; 2.0–4.5 min, 10–30%; 4.5–5.0 min, 30–100%; 5.0–6.3 min, 100%; 6.3–6.4 min, 100–0%; 6.4–8.0 min, 0%. The flow rate was 0.40 mL/min, the column temperature was maintained at 40 °C, and the injection volume was 3 μL. Mass spectrometric data were acquired in both positive- and negative-ion modes over an m/z range of 70–1050. The ion spray voltages were 3500 V in positive mode and −3000 V in negative mode. The sheath gas and auxiliary gas were set at 50 and 13 arb, respectively, the ion source temperature was 450 °C, and stepped collision energies of 20, 40, and 60 V were used. Raw LC–MS data were processed using Progenesis QI software for peak detection, peak extraction, retention time correction, and peak alignment. Metabolite annotation was performed based on the Human Metabolome Database (HMDB) [26], METLIN [27], and Majorbio’s in-house database. The resulting data matrix was uploaded to the Majorbio Cloud Platform for subsequent analysis. Metabolic features were filtered according to the 80% rule, whereby variables with non-zero values in at least 80% of the samples in at least one group were retained. Missing values were imputed using the minimum value in the raw data matrix, followed by sum normalization. Variables with a relative standard deviation greater than 30% in QC samples were removed, and the resulting data were log10-transformed prior to downstream statistical analysis.

Principal component analysis (PCA) and orthogonal partial least squares discriminant analysis (OPLS-DA) were performed using the ropls package in R (version 1.6.2), and model stability and the risk of overfitting were assessed by 200 permutation tests [28]. Differentially abundant metabolites (DAMs) were identified based on the variable importance in projection (VIP) scores derived from the OPLS-DA model and the results of Student’s *t*-test, using thresholds of VIP > 1.0 and *p* < 0.05. DAMs were subsequently subjected to pathway annotation and enrichment analysis based on the Kyoto Encyclopedia of Genes and Genomes (KEGG) database [29].

### 2.5. Crude Fat Content and Fatty Acid Composition Analysis

Crude fat content in muscle was determined using the Soxhlet extraction method. Muscle samples were freeze-dried to constant weight, ground, and extracted with petroleum ether in a Soxhlet apparatus. After extraction, the solvent was removed, and the lipid extract was dried to constant weight. Crude fat content was calculated gravimetrically and expressed as a percentage of muscle dry weight (DW). Muscle fatty acid composition was determined according to a previously described method [30]. An appropriate amount of muscle tissue was placed in a flask and hydrolyzed with 8.3 mol/L hydrochloric acid at 70–80 °C in a water bath for 40 min, with intermittent shaking to ensure thorough mixing. After hydrolysis, the samples were cooled to room temperature and mixed with 95% ethanol, followed by lipid extraction using a mixture of diethyl ether and petroleum ether. The extracts were combined and evaporated to dryness under reduced pressure using a rotary evaporator to obtain the lipid extract.

The lipid extract was mixed with 2% sodium hydroxide in methanol and refluxed in a water bath at 80 ± 1 °C until the oil droplets disappeared. Subsequently, 15% boron trifluoride solution was added, and the mixture was refluxed for an additional 2 min to complete fatty acid methyl esterification. After cooling to room temperature, fatty acid methyl esters (FAMEs) were extracted with n-heptane, and saturated sodium chloride solution was added to facilitate phase separation. The upper organic phase was collected, dried over anhydrous sodium sulfate, and transferred to an autosampler vial. FAMEs were analyzed using an Agilent 7820A gas chromatograph (Agilent Technologies, Santa Clara, CA, USA) equipped with a flame ionization detector (FID) and a TRACE TR-FAME capillary column (60 m × 0.25 mm i.d., 0.25 μm film thickness; Thermo Fisher Scientific, USA). The oven temperature was initially maintained at 60 °C for 3 min, increased to 175 °C at 5 °C/min and held for 15 min, and then increased to 220 °C at 2 °C/min and held for 10 min. Nitrogen was used as the carrier gas at a flow rate of 1.2 mL/min, with a split ratio of 1:100. The injection volume was 1 μL, and both the injector and detector temperatures were maintained at 250 °C. A fatty acid methyl ester standard mixture and the sample solutions were analyzed separately. Individual fatty acids were identified by comparison of their retention times with those of the standards and quantified using an external standard method based on peak areas. Fatty acid composition was expressed as the relative percentage of total identified fatty acids.

### 2.6. Transcriptome Sequencing and Analysis

Seven muscle samples from each group were used for transcriptome sequencing. Total RNA was extracted from muscle tissue using the TRIzol method. RNA concentration and purity were assessed using a NanoDrop 2000 spectrophotometer (Thermo Fisher Scientific, Wilmington, DE, USA) and RNA integrity was evaluated by agarose gel electrophoresis and an Agilent 5300 Fragment Analyzer (Agilent Technologies, Santa Clara, CA, USA). RNA samples that met the quality requirements were used for library construction. mRNA was enriched using oligo(dT) magnetic beads, fragmented, and reverse-transcribed to synthesize double-stranded cDNA. Sequencing libraries were then constructed through end repair, A-tailing, adapter ligation, fragment selection, and PCR amplification. After quality assessment, the libraries were subjected to paired-end sequencing on an Illumina NovaSeq X Plus platform.

Raw sequencing reads were processed using fastp (version 0.23.4) to remove adapter sequences and low-quality reads, thereby generating clean reads [31]. The clean reads were then aligned to the common carp reference genome ASM1834038v1 (*Cyprinus carpio* isolate SPL01; NCBI RefSeq assembly GCF_018340385.1) using HISAT2 (version 2.2.1), and reference-guided transcript assembly was performed using StringTie (version 2.2.1) [32,33].

Gene expression levels were quantified using RSEM (version 1.3.3). TPM values were used to describe and visualize gene expression abundance across samples. Differential expression analysis between YC and NC groups was performed based on the raw read count matrix using DESeq2 (version 1.42.0), with the NC group as the reference. Differentially expressed genes (DEGs) were identified using the criteria of adjusted *p* value < 0.05 and |log_2_FC| ≥ 1 [34]. Functional annotation and enrichment analyses of DEGs were performed based on the Gene Ontology (GO) and Kyoto Encyclopedia of Genes and Genomes (KEGG) databases. GO enrichment analysis was conducted using GOATOOLS (version 1.4.4) with Fisher’s exact test [35]. KEGG pathway enrichment analysis was also performed using Fisher’s exact test, with adjusted *p* < 0.05 considered statistically significant [29].

### 2.7. Proteomic Analysis

Seven muscle samples from each group were used for DIA-based quantitative proteomic analysis. Frozen muscle samples were lysed in protein extraction buffer containing 8 M urea, 1% SDS, and protease inhibitors. After tissue homogenization, low-temperature sonication, and centrifugation, the supernatants were collected. Protein concentrations were determined using the bicinchoninic acid (BCA) assay, and protein quality was assessed by SDS-PAGE.

Equal amounts of protein from each sample were subjected to reduction, alkylation, and tryptic digestion. The resulting peptides were desalted, dried, and quantified before DIA mass spectrometry analysis. Peptides were separated using a Vanquish Neo (Thermo Fisher Scientific, Germering, Germany) liquid chromatography system and analyzed in DIA acquisition mode. Raw mass spectrometry data were processed using Spectronaut 19 for database searching and protein abundance was quantified using the MaxLFQ method. Proteomic data were uploaded to the Majorbio Cloud Platform for statistical analysis and functional annotation. Differentially abundant proteins (DAPs) between YC and NC group were identified based on fold change and statistical significance, with the screening criteria of fold change ≥ 2 or ≤0.5 and *p* < 0.05. DAPs were further subjected to KEGG pathway annotation and enrichment analysis [29].

### 2.8. Comparative Multi-Omics Pathway Analysis and Mapping

To investigate the molecular basis associated with the yellow muscle phenotype in common carp, transcriptomic, untargeted metabolomic, and DIA-based proteomic datasets were jointly analyzed by comparing pathway-level changes across the three omics layers and mapping DEGs, DAPs, and DAMs onto key pathways. For all omics datasets, differential directions were defined relative to the NC group, with increases and decreases indicating higher and lower levels, respectively, in YC than in NC. DEGs, DAMs, and DAPs between YC and NC muscle were first identified separately and subjected to KEGG pathway annotation and enrichment analysis. Pathways shared across the three omics datasets or showing related biological functions were then comparatively evaluated, with particular attention to pathways associated with carotenoid accumulation, lipid metabolism, fatty acid composition remodeling, lipid transport, and membrane lipid composition. Differential genes, proteins, and metabolites were subsequently mapped onto selected shared pathways to examine their distribution across key metabolic reactions and to identify candidate pathways and molecules associated with the yellow muscle phenotype.

### 2.9. qRT-PCR Analysis of Candidate Genes

To determine the relative expression levels of candidate genes in YC and NC muscle, qRT-PCR analysis was performed. Total RNA was extracted from muscle samples using the TRIzol method, and RNA concentration, purity, and integrity were assessed. Qualified RNA samples were reverse-transcribed into cDNA for subsequent qRT-PCR analysis. qRT-PCR reactions were prepared using TB Green^®^ Premix Ex Taq^™^ II (TaKaRa, Dalian, China) and amplified on a real-time PCR system. Seven biological replicates were included per group, with three technical replicates for each biological sample. Gene-specific primer sequences used for the candidate and reference genes are listed in Table S2. The *gapdh* and *rpl8* genes were used as internal reference genes. For each biological sample, the mean Ct value of *gapdh* and *rpl8* was used as the reference Ct, and the relative expression levels of candidate genes were calculated using the 2^−ΔΔCt^ method.

### 2.10. Statistical Analysis

Unless otherwise stated, quantitative data are presented as mean ± SD. Before statistical analysis, data for muscle color parameters, total carotenoid content, crude fat content, fatty acid composition, and qRT-PCR relative expression levels were tested for normality and homogeneity of variance. Differences between the YC and NC group were analyzed using independent-samples *t*-tests, with *p* < 0.05 considered statistically significant.

The relationship between total carotenoid content and b* values was evaluated using Pearson correlation analysis. To determine whether this association remained after accounting for phenotype group, an additional multiple linear regression analysis was performed with b* values as the dependent variable and total carotenoid content and phenotype group (YC/NC) as predictors. Pearson correlation coefficients were calculated among individual fatty acids and fatty acid classes to construct the correlation matrix. Mantel tests were used to evaluate the associations between total carotenoid content and individual fatty acid variables, with Mantel’s r representing association strength and Mantel’s *p* value indicating statistical significance. Pearson correlation analysis was used to assess the relationship between crude fat content and total carotenoid content. Correlation analyses were visualized using the ChiPlot online platform.

For qRT-PCR analysis, relative expression levels of candidate genes were calculated using the 2^−ΔΔCt^ method, with the mean Ct value of *gapdh* and *rpl8* used for normalization. Differences in relative gene expression between YC and NC were evaluated using independent-samples *t*-tests. Statistical analyses were primarily performed using SPSS software (version 27.0.1).

## 3. Results

### 3.1. Muscle Color Characteristics of YC and NC Group

**Figure 2:**
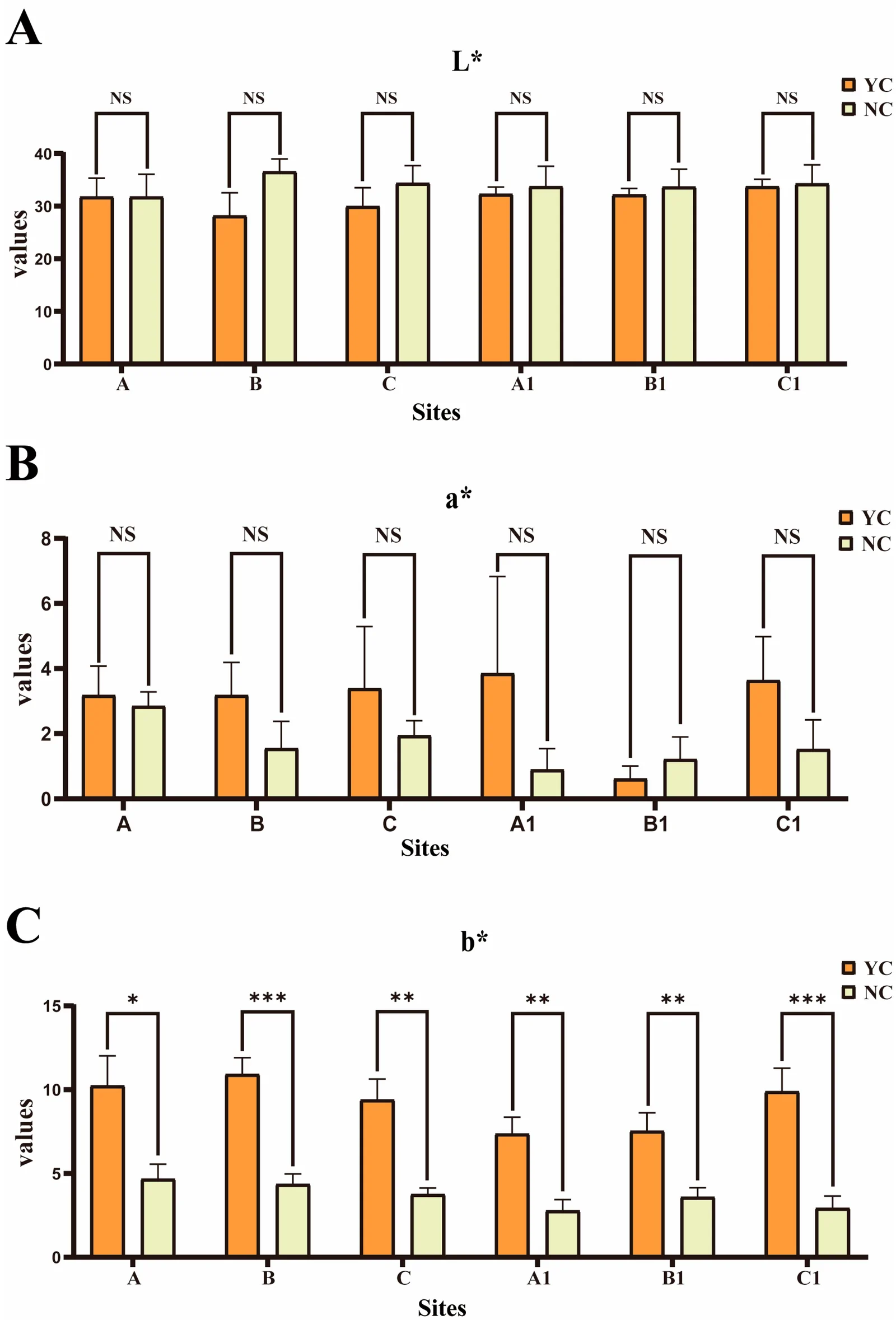
Comparison of muscle color parameters between Yellow muscle common carp (YC) and Normal muscle common carp (NC) at six measurement sites: (**A**) Lightness (L*); (**B**) redness (a*); (**C**) yellowness (b*). Data are presented as mean ± SD (*n* = 7 per group). Differences between YC and NC were assessed using an independent-samples *t*-test. NS, not significant (*p* > 0.05); *, *p* < 0.05; **, *p* < 0.01; ***, *p* < 0.001.

To characterize muscle color differences between YC and NC group, the CIELAB color parameters (L*, a*, b*) were measured in muscle samples from both groups. At the six measurement sites (A, B, C, A1, B1, and C1), no significant differences were detected in the L* or a* values between the YC group and the NC group (*p* > 0.05) (Figure 2A,B). In contrast, b* values were significantly higher in YC muscle than in NC muscle at all measurement sites (*p* < 0.05) (Figure 2C). Thus, the muscle color difference between YC and NC group was primarily characterized by enhanced b* value, with no significant differences in L* values or a* values.

### 3.2. Relationship Between Carotenoid Accumulation and the Yellow Muscle Phenotype

**Figure 3:**
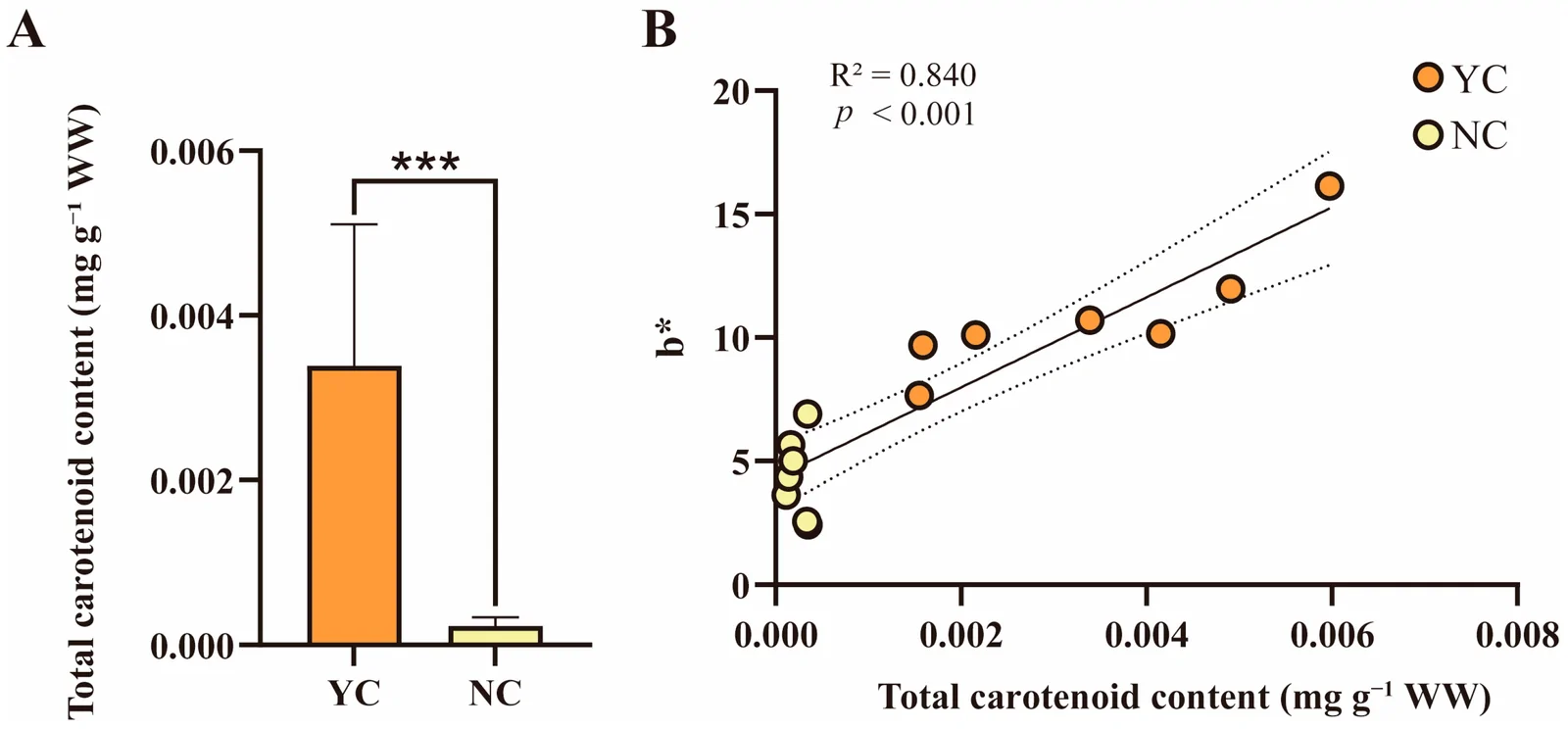
Relationship between total carotenoid content and the yellow muscle phenotype in common carp: (**A**) Total carotenoid content in Yellow muscle common carp (YC) and Normal muscle common carp (NC) muscle. Data are presented as mean ± SD (*n* = 7 per group). ***, *p* < 0.001. (**B**) Relationship between total carotenoid content and the b* value measured at the corresponding B site, assessed by Pearson correlation analysis (*n* = 14). Total carotenoid content was significantly positively correlated with the B-site b* value (R^2^ = 0.840, *p* < 0.001). Total carotenoid content was expressed on a wet-weight (WW) basis. The solid line represents the fitted regression line, and the dashed lines indicate the 95% confidence bands.

To assess whether the yellow muscle phenotype is associated with carotenoid accumulation, total carotenoid content was measured in muscle tissue from the YC and NC group. The results showed that the total carotenoid content in the YC group was significantly higher than that in the NC group (*p* < 0.001) (Figure 3A). Pearson correlation analysis further indicated a significant positive correlation between total carotenoid content and the b* value of muscle (*p* < 0.001) (Figure 3B). To account for the potential effect of phenotype group, an additional multiple linear regression analysis was performed. After adjustment for phenotype group, total carotenoid content remained significantly associated with muscle b* values (*p* = 0.004), and the overall model explained 87.2% of the variation in b* value (R^2^ = 0.872). Taken together, these results indicate a close association between total carotenoid content and muscle yellowness, which remained significant after accounting for phenotype group.

### 3.3. Untargeted Metabolomic Analysis Reveals Metabolic Differences Between YC and NC Muscle

**Figure 4:**
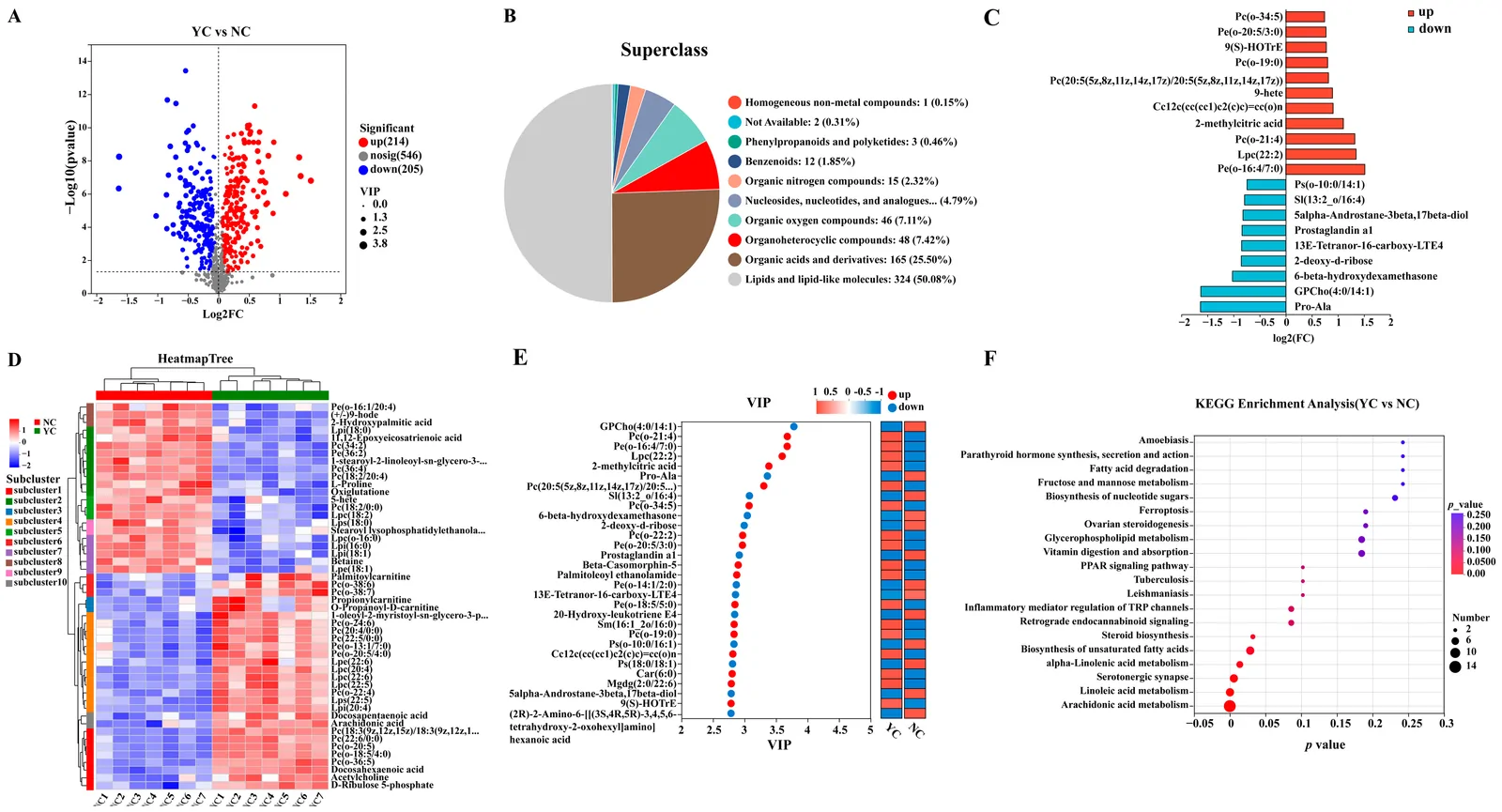
Untargeted metabolomic analysis of Yellow muscle common carp (YC) and Normal muscle common carp (NC) muscle. (**A**) Volcano plot of DAMs between YC and NC; (**B**) pie chart of DAMs classified by superclass; (**C**) bar plot of selected DAMs based on log_2_FC; (**D**) clustered heatmap of DAMs; (**E**) VIP score plot of DAMs; (**F**) bubble plot of KEGG pathway enrichment analysis.

To investigate the metabolic basis associated with the difference in muscle b* values between YC and NC, untargeted metabolomic analysis was performed on muscle samples from both groups. Using NC as the control group, a total of 419 DAMs were identified, of which 214 DAMs showed significantly higher relative abundance and 205 DAMs showed significantly lower relative abundance in YC (Figure 4A). Classification of the DAMs showed that they were mainly assigned to lipids and lipid-like molecules, organic acids and derivatives, and other metabolite classes. Among these, lipids and lipid-like molecules constitute the largest category, accounting for 50.08% of all DAMs (Figure 4B).

Among the identified DAMs, various phospholipids (including PC and PE) and arachidonic acid (AA) metabolism-related metabolites (such as prostaglandins) were enriched in YC samples (Figure 4C,E). Heatmap visualization and hierarchical clustering analysis of the DAMs revealed distinct metabolic profiles between YC and NC samples, with good consistency among biological replicates within each group (Figure 4D). KEGG pathway enrichment analysis revealed that DAMs were primarily enriched in pathways related to lipid metabolism, including AA metabolism, linoleic acid metabolism, and the biosynthesis of unsaturated fatty acids (Figure 4F). Additionally, in the untargeted metabolomics dataset, the relative abundances of two carotenoids, canthaxanthin and α-cryptoxanthin, were significantly higher in the YC group than in the NC group (Table S1). Overall, the metabolic differences between the YC and NC muscle involved both increased abundance of characteristic carotenoids and extensive alterations in lipid metabolism.

### 3.4. Differences in Muscle Fatty Acid Composition Between YC and NC Group

To further elucidate differences in muscle fatty acid composition between the YC group and the NC group, the fatty acid profiles of muscle samples from both groups were analyzed. The results revealed significant differences between the YC group and the NC group in the proportions of various fatty acids and fatty acid categories (Table 1). Specifically, the concentrations of the saturated fatty acids (SFA) C14:0, C16:0, C18:0, and C20:0 were all significantly higher in the YC group than in the NC group. Furthermore, the total SFA proportion in the YC group (42.76 ± 1.34%) was also significantly higher than that in the NC group (24.15 ± 0.99%).

In the YC group, the proportions of the monounsaturated fatty acids (MUFA) C14:1n-5, C16:1n-7, and C20:1n-9 were significantly higher than those in the NC group, while the proportion of C18:1n-9 was significantly lower in the YC group than in the NC group. Although there were differences in the composition of various MUFA, there was no significant difference in total MUFA content between the YC group (36.46 ± 3.24%) and the NC group (38.37 ± 1.59%).

The proportion of total polyunsaturated fatty acids (PUFA) in the YC group (20.77 ± 2.37%) was significantly lower than that in the NC group (37.49 ± 0.66%). Specifically, the proportion of the PUFA C18:2n-6 in the YC group was significantly lower than that in the NC group, which was one of the main reasons for the difference in total PUFA content between the two groups. Consistent with this, the total proportion of n-6 PUFA in the YC group (11.53 ± 2.46%) was significantly lower than that in the NC group (36.60 ± 0.55%).

Among n-3 PUFA, the proportions of C18:3n-3, C20:5n-3, and C22:6n-3 in the YC group were all significantly higher than those in the NC group. The total proportion of n-3 PUFA in the YC group (9.25 ± 0.64%) was also significantly higher than that in the NC group (0.89 ± 0.12%). In addition, the n-3/n-6 ratio in the YC group was significantly higher than that in the NC group.

**Figure 5:**
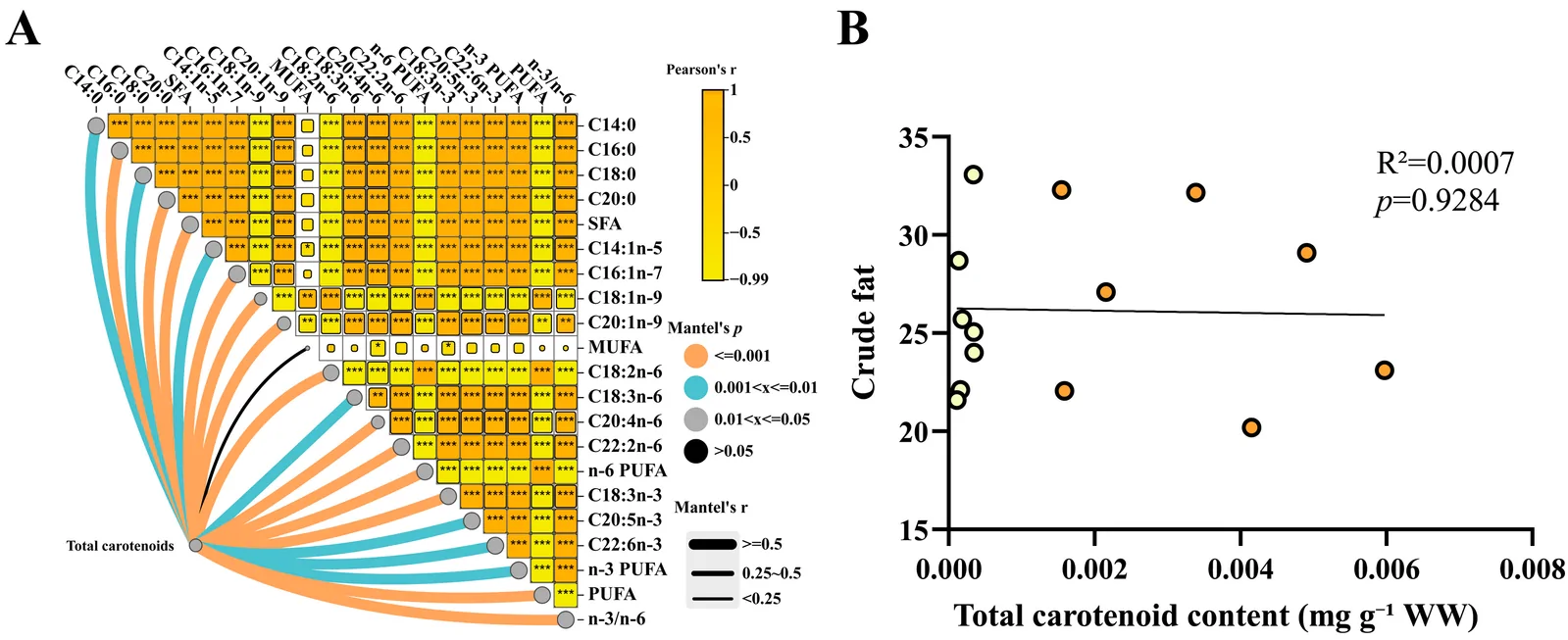
Correlations among total carotenoid content, fatty acid composition, and crude fat content: (**A**) Correlation network between total carotenoid content and muscle fatty acid composition. The matrix in the upper right shows pairwise Pearson correlations among individual fatty acids and fatty acid classes. The color scale represents the correlation coefficients, and asterisks indicate statistical significance. The connecting lines on the left represent Mantel test results between total carotenoid content and individual fatty acid variables. Line color represents Mantel’s *p* value, whereas line width represents Mantel’s r value. *, *p* < 0.05; **, *p* < 0.01; ***, *p* < 0.001. (**B**) Pearson correlation analysis between crude fat content and total carotenoid content. Each point represents an individual sample, with orange indicating Yellow muscle common carp (YC) and light yellow indicating Normal muscle common carp (NC). The R^2^ and *p* values are shown in the plot. The solid line represents the fitted regression line. SFA, saturated fatty acids; MUFA, monounsaturated fatty acids; PUFA, polyunsaturated fatty acids; n-3 PUFA, n-3 polyunsaturated fatty acids; n-6 PUFA, n-6 polyunsaturated fatty acids.

There was no significant difference in crude fat content between the YC group and the NC group (Table 1). To further investigate the relationship among total carotenoid content, fatty acid composition, and crude fat content, a correlation analysis was conducted on these three variables. The Mantel test indicated a significant association between total carotenoid content and several fatty acid variables, while Pearson correlation analysis revealed varying degrees of correlation among individual fatty acids and fatty acid categories (Figure 5A). In contrast, no significant Pearson correlation was observed between crude fat content and total carotenoid content (Figure 5B).

Overall, these results indicate that the lipid differences between YC and NC were primarily reflected in fatty acid composition rather than in overall crude fat accumulation. There were significant differences in muscle fatty acid composition between the YC group and the NC group, primarily characterized by higher proportions of SFA and n-3 PUFA in the YC group, and lower proportions of n-6 PUFA and total PUFA.

### 3.5. Transcriptomic Analysis Reveals Gene Expression Differences Between YC and NC Muscle

**Figure 6:**
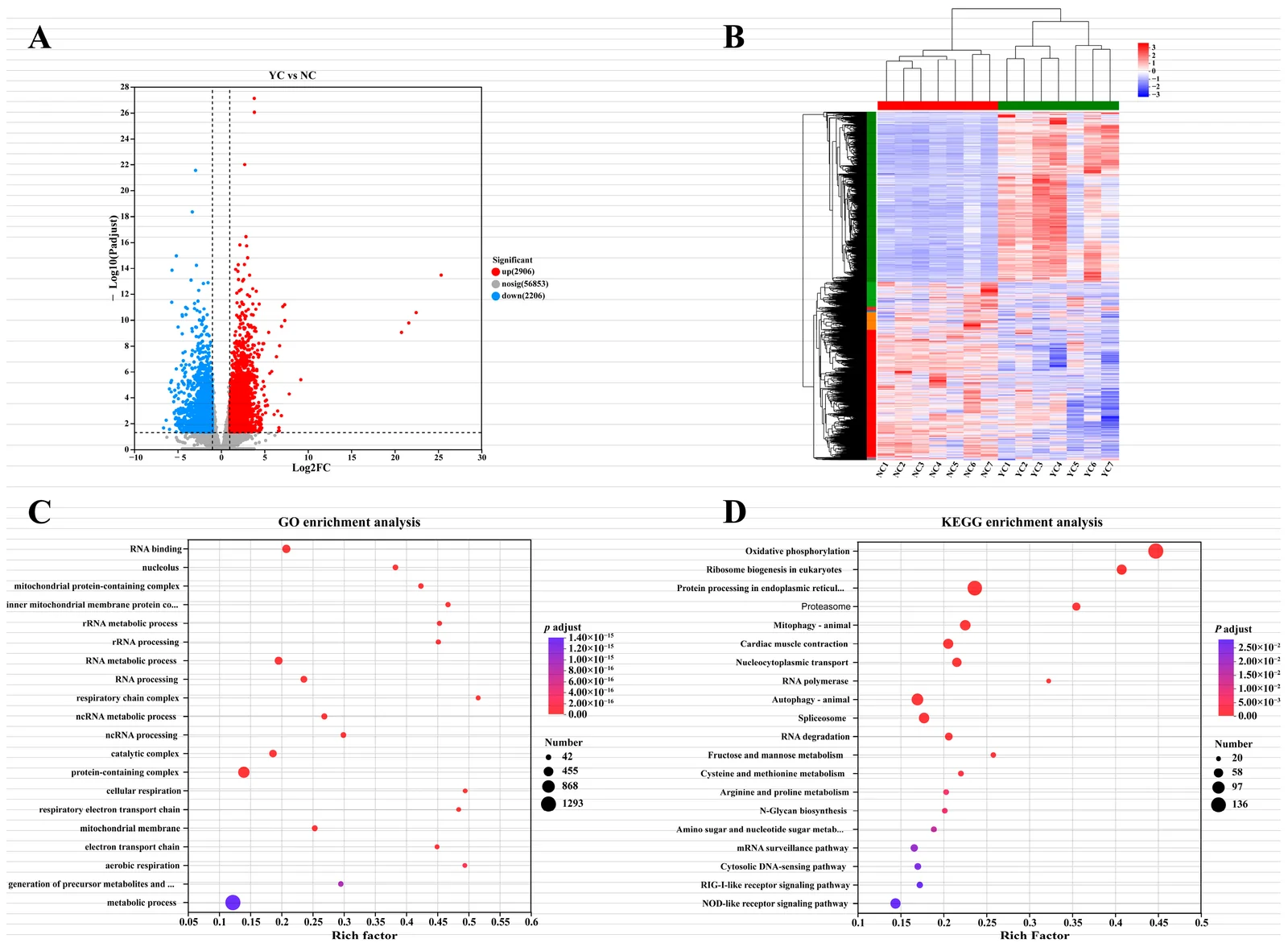
Transcriptomic analysis of Yellow muscle common carp (YC) and Normal muscle common carp (NC) muscle: (**A**) Volcano plot of DEGs, with red indicating upregulated genes, blue indicating downregulated genes, and gray indicating genes without significant differences; (**B**) hierarchical clustering heatmap of DEGs; (**C**) bubble plot of GO functional enrichment analysis, where the rich factor represents the degree of enrichment, bubble size represents the number of genes, and color represents the adjusted *p* value; (**D**) bubble plot of KEGG pathway enrichment analysis.

To further characterize the molecular differences associated with the muscle phenotype, transcriptomic sequencing was performed on muscle samples from both groups. A total of 5112 DEGs were identified, with 2906 upregulated genes and 2206 downregulated genes in the YC group (Figure 6A). Hierarchical cluster analysis revealed distinct gene expression patterns between the YC and NC group, and the biological replicates within each group showed good consistency (Figure 6B).

GO enrichment analysis revealed that the DEGs were mainly enriched in RNA-related terms, including RNA binding, nucleolus, rRNA metabolic process, and RNA processing, as well as terms related to energy metabolism, involving mitochondrial protein complexes, respiratory chain complexes, and the electron transport chain (Figure 6C). These results indicate broad differences between YC and NC group in RNA-related processes and energy metabolism-associated functions. KEGG pathway enrichment analysis showed that the DEGs were mainly enriched in pathways related to energy metabolism and fundamental cellular processes, such as oxidative phosphorylation, ribosome biogenesis, protein processing in the endoplasmic reticulum, proteasome, and mitophagy (Figure 6D).

### 3.6. Proteomic Analysis Reveals Changes in Lipid Metabolism-Related Proteins

**Figure 7:**
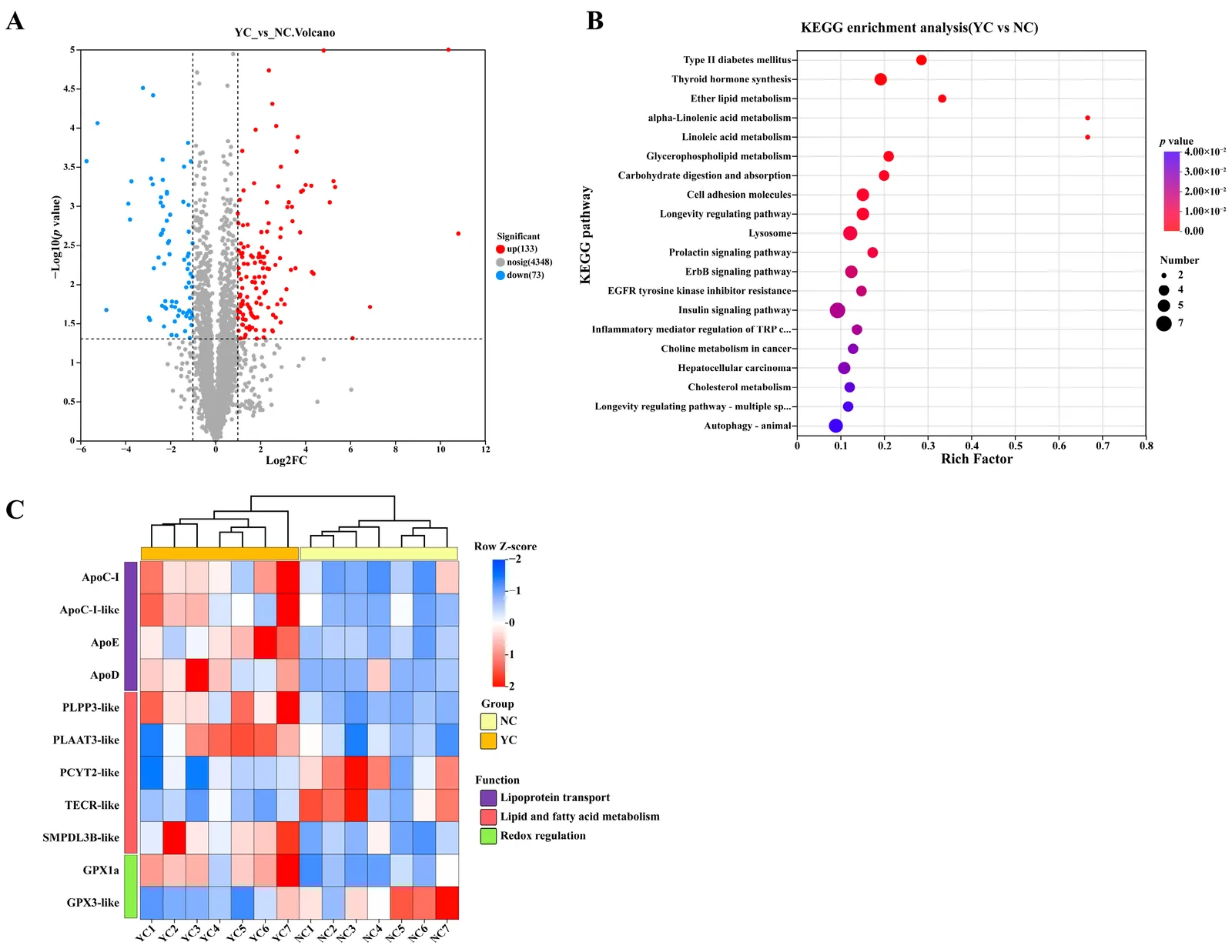
Proteomic analysis of Yellow muscle common carp (YC) and Normal muscle common carp (NC) muscle: (**A**) Volcano plot of DAPs, with red indicating increased abundance, blue indicating decreased abundance, and gray indicating no significant difference; (**B**) bubble plot of KEGG pathway enrichment analysis, where the rich factor represents the degree of enrichment, bubble size represents the number of proteins, and color represents the *p* value; (**C**) clustered heatmap of DAPs associated with lipoprotein transport, lipid and fatty acid metabolism, and redox regulation. Protein abundances were log_2_-transformed and subsequently standardized by row-wise Z-score, followed by hierarchical clustering of samples. Red and blue indicate relatively higher and lower protein abundance, respectively.

To further characterize differences between the YC and NC muscle at the protein level, DIA-based quantitative proteomic analysis was performed on samples from both groups. Using NC as the control group, a total of 206 DAPs were identified, of which 133 DAPs showed significantly higher abundance and 73 DAPs showed significantly lower abundance in YC (Figure 7A). KEGG pathway enrichment analysis revealed that the DAPs were mainly enriched in lipid metabolism-related pathways, including glycerophospholipid metabolism, α-linolenic acid metabolism, linoleic acid metabolism, and ether lipid metabolism. Among these pathways, α-linolenic acid metabolism and linoleic acid metabolism showed relatively high rich factors but involved relatively few DAPs, whereas glycerophospholipid metabolism included a larger number of DAPs (Figure 7B).

Subsequently, hierarchical clustering analysis was performed on selected DAPs associated with lipoprotein transport, lipid and fatty acid metabolism, and redox regulation. YC and NC samples clustered into two distinct branches, indicating significant differences between the two groups in the abundance patterns of these proteins (Figure 7C). Among proteins associated with lipoprotein transport, ApoC-I, ApoC-I-like, ApoE, and ApoD proteins showed higher abundance in YC. Among proteins involved in lipid and fatty acid metabolism, PLPP3-like, PLAAT3-like, and SMPDL3B-like proteins showed higher abundance in YC, whereas PCYT2-like and TECR-like proteins showed lower abundance. For redox-related proteins, GPX1a showed higher abundance in YC, whereas GPX3-like showed lower abundance. These results indicate that the yellow muscle phenotype is accompanied by lipid metabolism and lipoprotein transport.

### 3.7. Comparative Pathway Analysis and Mapping Across Three Omics Datasets

**Figure 8:**
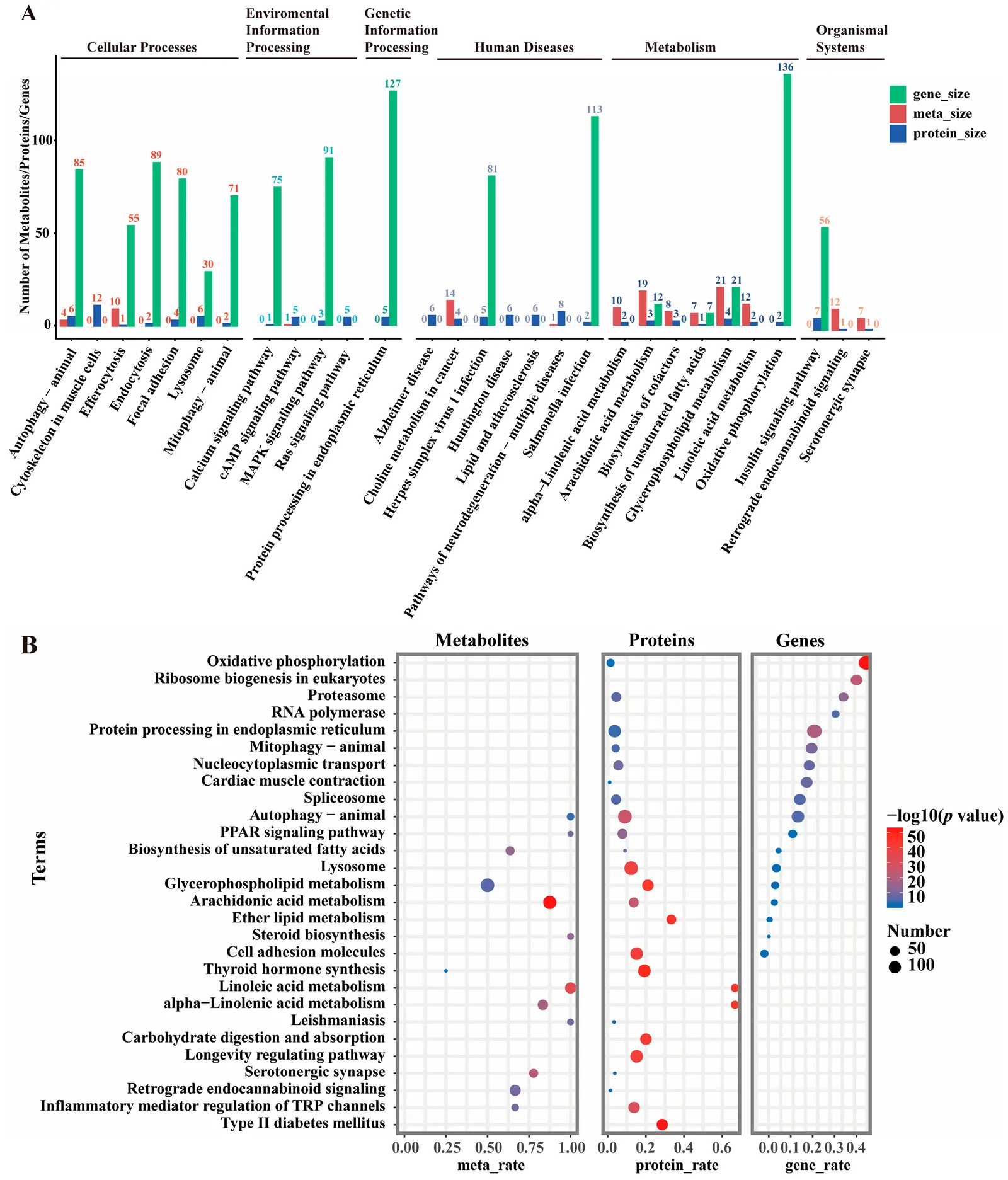
Comparative KEGG pathway analysis across three omics datasets: (**A**) KEGG functional classification of DEGs, DAPs, and DAMs. The *x*-axis represents KEGG level 2 functional categories, and the *y*-axis represents the number of differential molecules identified in each omics dataset. Green, red, and blue bars represent the numbers of DEGs, DAMs, and DAPs, respectively. (**B**) KEGG pathway enrichment analysis across the three omics datasets. Metabolomic, proteomic, and transcriptomic results are shown from left to right, respectively. The *x*-axis represents the proportion of differential molecules among all molecules annotated to each pathway, and the *y*-axis represents KEGG pathway names. Bubble size represents the number of differential molecules mapped to each pathway, whereas bubble color represents enrichment significance [−log10 (*p* value)], with darker red indicating greater statistical significance.

Comparative analysis of KEGG pathways identified three common lipid metabolism-related pathways across the transcriptomic, proteomic, and metabolomic datasets: glycerophospholipid metabolism, biosynthesis of unsaturated fatty acids, and AA metabolism (Figure 8A). Therefore, these common pathways were selected for subsequent pathway mapping. Comparison of the KEGG enrichment profiles across the three omics datasets revealed distinct enrichment patterns (Figure 8B). The transcriptomic dataset was mainly enriched in pathways related to fundamental cellular processes, such as oxidative phosphorylation and ribosome biogenesis, whereas lipid metabolism-related pathways were more prominent in the metabolomic and proteomic datasets. In the metabolomic dataset, AA metabolism, linoleic acid metabolism, and α-linolenic acid metabolism exhibited relatively high enrichment ratios and strong enrichment signals. Glycerophospholipid metabolism was enriched in all three omics datasets, with a relatively large number of DAMs mapped to this pathway in the metabolomic dataset.

DEGs, DAPs, and DAMs were further mapped onto the KEGG reference pathways of glycerophospholipid metabolism, AA metabolism, and biosynthesis of unsaturated fatty acids (Figure S1). Differential molecules from the three omics approaches were distributed across multiple reaction nodes within these pathways. In the glycerophospholipid metabolism, the mapped molecules mainly included phospholipids (such as PC and PE) and their associated metabolic enzymes. AA metabolism primarily involved AA and its downstream oxidized lipid metabolites, whereas the biosynthesis of unsaturated fatty acids mainly involved molecules associated with fatty acid desaturation, elongation, and the formation of long-chain polyunsaturated fatty acids. These pathway maps illustrated the distribution and correspondence of differential molecules from the three omics levels within key lipid metabolic pathways. Collectively, the multi-omics comparison converged on alterations in glycerophospholipid metabolism, fatty acid metabolism, and related lipid pathways in YC muscle.

### 3.8. qRT-PCR Expression Analysis of Candidate Genes

**Figure 9:**
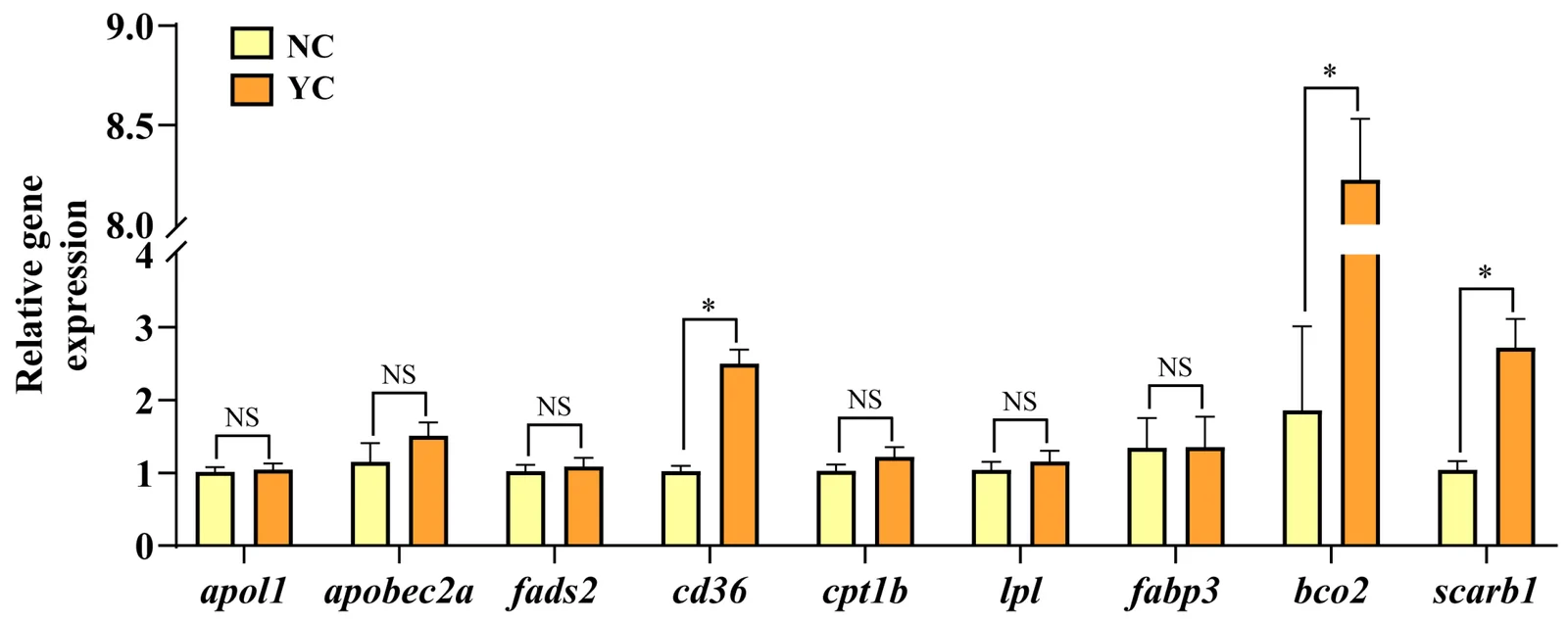
Relative mRNA expression levels of candidate genes in YC and NC muscle: The expression levels of candidate genes related to carotenoid metabolism, lipid transport, and fatty acid metabolism were determined by qRT-PCR. Relative expression levels were calculated using the 2^−ΔΔCt^ method and normalized using *gapdh* and *rpl8* as internal reference genes. Data are presented as mean ± SD (*n* = 7 per group). Differences between Yellow muscle common carp (YC) and Normal muscle common carp (NC) were assessed using an independent-samples *t*-test. *, *p* < 0.05; NS, not significant (*p* > 0.05).

To further evaluate the relationship between the yellow muscle phenotype in common carp and carotenoid metabolism, nine candidate genes associated with carotenoid metabolism, lipid transport, and fatty acid metabolism were selected for qRT-PCR analysis. Among these genes, CD36 molecule (*cd36*), β-carotene oxygenase 2 (*bco2*), and scavenger receptor class B member 1 (*scarb1*) were significantly upregulated in YC relative to NC (*p* < 0.05) (Figure 9). These results indicate that the transcriptional levels of genes associated with lipid transport and carotenoid metabolism are significantly upregulated in YC muscle. In addition, no significant differences were observed for apolipoprotein L1 (*apol1*), apolipoprotein B mRNA editing enzyme, catalytic polypeptide-like 2a (*apobec2a*), fatty acid desaturase 2 (*fads2*), lipoprotein lipase (*lpl*), carnitine palmitoyltransferase 1b (*cpt1b*), and fatty acid binding protein 3 (*fabp3*) (*p* > 0.05) (Figure 9). These results indicate that genes associated with lipid transport and carotenoid metabolism are upregulated in the muscle of the YC group, and these genes may be involved in the regulatory process of muscle color yellowness.

## 4. Discussion

### 4.1. Enhanced Yellowness Is the Primary Characteristic of the YC Muscle Phenotype

The present study showed that the difference in muscle color between YC and NC was characterized by a significant increase in b* values, whereas L* and a* values did not differ significantly between the two groups. This indicates that the YC phenotype does not represent a broad alteration across multiple dimensions of muscle color, but rather a distinct color phenotype primarily characterized by enhanced yellowness.

Changes in fish muscle color appearance can involve multiple CIELAB parameters simultaneously. For example, in another study on common carp, both a* and b* values of the fillets increased, resulting in an overall shift toward a more yellow-red hue [36]. Similarly, increases in both a* and b* values have been reported in *Salmo salar*, whereas a red fillet defect in *Ictalurus punctatus* was associated with increases in a* and b* together with a decrease in L* [37,38]. These findings illustrate that changes in fish muscle coloration may involve coordinated shifts in multiple color dimensions.

In contrast to the multi-parameter color changes described above, studies on some fish species have reported that muscle color differences are primarily reflected by changes in the b* value. In a pigmentation dose-response experiment in *Clarias macrocephalus*, no significant differences were observed in muscle L* or a* values among the treatment groups, whereas b* values increased significantly [39]. A similar pattern was observed in *Micropterus salmoides*, *Pangasianodon hypophthalmus*, and hybrid grouper, where an increase in muscle yellowness was not accompanied by significant changes in L* or a* values [40,41,42].

These findings are consistent with the results of the present study and further indicate that coordinated changes in a single (b*) or multiple (a*, b*, and L*) color dimensions may account for perceptible changes in fish muscle color. In this study, increased yellowness was the defining color characteristic of the YC muscle phenotype, prompting us to further investigate the pigmentary factors associated with this coloration.

### 4.2. Carotenoid Accumulation Provides the Pigmentary Basis for the Yellow Muscle Phenotype

In the present study, total carotenoid content was significantly higher in YC muscle than in NC muscle and was significantly positively correlated with b* values, indicating that carotenoid accumulation was closely associated with the enhanced yellowness of YC muscle. Carotenoids are important pigments in fish tissues, and their deposition can directly influence muscle coloration. Previous studies have shown that increased carotenoid accumulation is generally associated with enhanced yellow, orange, or red coloration in fish tissues [20,42,43]. These findings support the notion that increased carotenoid accumulation is a key pigmentary characteristic contributing to the enhanced yellowness of YC muscle.

The specific coloration of fish muscle is determined not only by total carotenoid abundance, but also by carotenoid composition and the relative proportions of individual carotenoid species [44,45]. Different carotenoid species exhibit distinct light absorption characteristics due to variations in their conjugated double-bond structures, resulting in tissue coloration ranging from yellow to red [24,46,47]. Among these pigments, ketocarotenoids such as astaxanthin and canthaxanthin generally impart red to orange-red coloration and can contribute to both the redness and yellowness of fish tissues [43]. In contrast, pigments such as lutein, zeaxanthin, and cryptoxanthin tend to impart yellow to yellow-orange hues to tissues [24,47,48]. Thus, the enhanced yellowness observed in YC muscle reflects not only greater carotenoid accumulation but also a distinct carotenoid composition.

Untargeted metabolomic analysis revealed significantly higher relative abundances of canthaxanthin and α-cryptoxanthin in YC muscle. Taken together, our results suggest that carotenoid accumulation is closely associated with canthaxanthin and α-cryptoxanthin potentially representing important carotenoids associated with the yellow muscle phenotype.

### 4.3. Association of Fatty Acid Composition Remodeling and Lipid Metabolic Changes with Carotenoid Accumulation

In the present study, there was no significant difference in crude fat content between the YC and NC muscle, and no significant correlation was observed between crude fat content and total carotenoid content. These findings indicate that the higher carotenoid accumulation in YC muscle cannot be simply attributed to increased crude fat deposition. Instead, marked differences in muscle fatty acid composition were observed between the two groups, suggesting that the lipid differences associated with the YC phenotype are primarily reflected in fatty acid composition rather than in the overall accumulation of crude fat.

Previous studies in fish suggest that carotenoid deposition is significantly influenced by lipid composition. In Arctic charr (*Salvelinus alpinus*), dietary lipid level and astaxanthin supplementation jointly affected muscle and skin pigmentation [21]. Similarly, in Atlantic salmon (*Salmo salar*), dietary EPA and DHA levels were associated with astaxanthin deposition in muscle, with DHA showing a relatively strong positive relationship with muscle astaxanthin concentration [19]. Taken together, these findings indicate that differences in fatty acid composition may be relevant to deposition and retention of carotenoids in fish muscle.

Among the altered fatty acids, EPA and DHA were particularly noteworthy because both were present at higher proportions in YC muscle and showed significant associations with total carotenoid content in the Mantel analysis. Although these results do not demonstrate that EPA or DHA directly promotes carotenoid deposition, their higher proportions in YC muscle together with their significant associations with total carotenoid content identify them as candidate fatty acid features associated with carotenoid accumulation.

Another noteworthy feature of the fatty acid profile was the increase in AA in YC muscle, despite the overall lower proportion of n-6 PUFA. This contrasting pattern suggests that the n-6 PUFA difference between YC and NC reflects compositional redistribution rather than a uniform decrease. Importantly, the increase in AA content was consistent with the enrichment of AA metabolism in the metabolomic analysis and aligns with its identification as a common lipid-related pathway across the three omics datasets. Given that AA is an essential component of membrane lipids and a precursor of multiple lipid signaling molecules [49,50,51], the convergence of fatty acid profiling and multi-omics results highlights AA metabolism as another lipid feature worthy of attention in the YC muscle phenotype. However, *fads2* expression did not differ significantly between YC and NC, indicating that the higher AA proportion cannot be attributed to enhanced FADS2-mediated desaturation based on the present data. Accordingly, the fatty acid remodeling described here refers to the overall differences in fatty acid composition rather than evidence of increased FADS2-driven desaturation.

Changes in glycerophospholipid-related molecules (such as PC and PE) indicate that membrane lipid composition may also differ between YC and NC muscle. Glycerophospholipids are major structural components of cellular membranes, and changes in their composition and fatty acid constituents can influence lipid packing, membrane fluidity, and the local hydrophobic environment [52,53,54]. These properties are particularly relevant to carotenoids, as their localization and orientation within membranes are influenced by the surrounding lipid composition [46,55,56]. Taken together, these findings suggest that alterations in fatty acid and membrane lipid composition may alter the membrane properties associated with the localization, distribution, and retention of carotenoids in YC muscle.

Since fish cannot synthesize carotenoids de novo, their accumulation in muscle depends on exogenous acquisition, followed by transport and tissue deposition [57,58,59]. Carotenoids are highly lipophilic molecules, and their absorption and tissue distribution are closely linked to lipid-mediated transport processes [60]. In fish, apolipoproteins have been identified as serum lipoproteins carrying carotenoids [13]. Furthermore, studies in Atlantic salmon showed that astaxanthin is transported in plasma lipoprotein fractions, with changes in lipid availability altering its distribution in circulating lipoproteins [61]. Consistent with these findings, our proteomic analysis showed higher abundances of ApoC-I, ApoC-I-like, ApoE, and ApoD in YC muscle. Combined with the remodeling of fatty acid and membrane lipid composition, these changes in apolipoprotein levels provide protein-level evidence that the greater accumulation of carotenoids in YC muscle is accompanied by changes in lipoprotein-related processes.

It should also be noted that the transcriptomic changes were mainly associated with energy metabolism, RNA-related processes, and protein synthesis, rather than showing a predominantly lipid-metabolic pattern. These changes may instead represent broader or secondary physiological responses associated with the YC phenotype rather than direct drivers of yellow muscle formation.

### 4.4. Proposed Working Model for Enhanced Muscle Coloration in Common Carp

**Figure 10:**
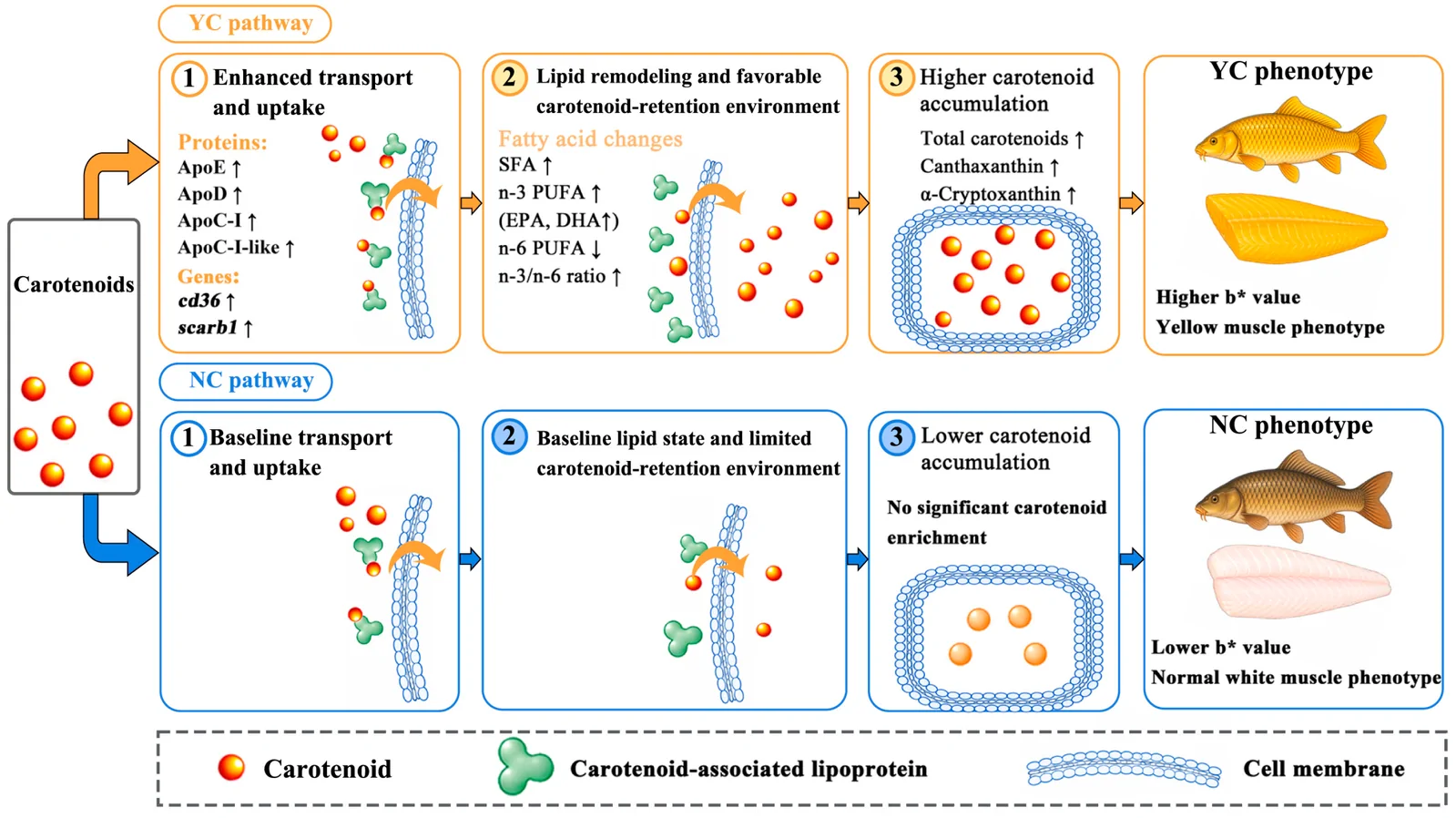
Proposed working model for enhanced muscle coloration in common carp: The left panel shows candidate factors related to lipid and lipophilic molecule transport, including apolipoproteins, *scarb1*, and *cd36*. The middle panel illustrates the remodeling of fatty acid composition, characterized mainly by unchanged crude fat content, increased SFA, n-3 PUFA, EPA, DHA, and the n-3/n-6 ratio, along with a decreased n-6 PUFA. The right panel shows the accumulation of carotenoids in muscle, including increased total carotenoid content, canthaxanthin, and α-cryptoxanthin, accompanied by upregulation of the expression of *bco2*, a candidate gene associated with carotenoid metabolism. Dashed arrows indicate putative relationships or possible feedback regulation. These features are associated with a yellow muscle phenotype characterized by increased muscle yellowness (higher b* values). ↑ indicates upregulation or increase; ↓ indicates downregulation or decrease.

Based on a comprehensive analysis of phenotypic, compositional, and multi-omics evidence, we propose a working model for the yellow muscle phenotype of common carp. In YC muscle, the higher abundances of ApoE, ApoD, ApoC-I, and ApoC-I-like, together with the increased expression of *cd36* and *scarb1*, suggest alterations in lipoprotein-associated transport and the handling of lipids and other lipophilic compounds. These changes are accompanied by higher total carotenoid content and increased relative abundances of canthaxanthin and α-cryptoxanthin, ultimately corresponding to the higher b* value and yellow muscle phenotype observed in YC (Figure 10).

qRT-PCR results provide additional evidence of differences in processes related to lipid uptake and carotenoid metabolism in YC muscle. The significant upregulation of *cd36* and *scarb1* in YC muscle suggests differences in cellular processes related to the uptake and handling of lipids and other lipophilic compounds. SCARB1 has been shown to mediate the uptake of β-carotene and cholesterol, supporting its potential relevance to carotenoid-associated transport [62]. In addition, *bco2* was significantly upregulated in YC muscle. BCO2 is a carotenoid-cleaving enzyme that participates in the oxidative metabolism of carotenoids, and variation in *bco2* expression has been associated with carotenoid pigmentation in fish [63,64]. The elevated *bco2* expression in YC muscle may represent a metabolic response to the higher carotenoid content, reflecting increased carotenoid turnover rather than directly promoting carotenoid deposition [65].

Since the experimental fish were wild-caught, potential variation in genetic background, age, sex, maturity stage, and feeding history before capture could not be fully controlled. Although all fish were maintained under standardized laboratory conditions for 15 days before sampling, this period may have reduced short-term environmental variation but could not eliminate the effects of previous diet, including differences in carotenoid intake. Therefore, the molecular and metabolic differences identified in the present study should be interpreted as characteristics associated with the yellow muscle phenotype rather than as definitive evidence of exclusively intrinsic causal mechanisms.

## 5. Conclusions

Muscle color is an important quality indicator for evaluating aquatic product quality, and studying the formation of muscle color in fish is of great significance. This study aimed to elu-cidate the pigment composition, metabolic characteristics, and related biological processes in-volved in the formation of the yellow muscle phenotype in common carp, thereby further revealing the biological basis of muscle color variation in common carp. In this study, the muscle b* values in the YC group was significantly higher than that in the NC group, while there were no significant differences in L* values and a* values. The YC group had higher total carotenoid content, which was positively correlated with the b* value. The relative abundances of canthaxanthin and α-cryptoxanthin were higher in YC muscle, further indicating that carotenoids are key to the enhanced yellowness of the flesh. Although crude fat content remained unchanged, the fatty acid composition in the YC group underwent significant restructuring, characterized by higher proportions of SFA and n-3 PUFA (including EPA and DHA), while the proportions of n-6 PUFAs and total PUFAs were lower. Mult-omics analysis further indicated that coordinated changes occurred in glycerophospholipid metabolism, unsaturated fatty acid biosynthesis, arachidonic acid metabolism, and lipid- and lipoprotein-related processes. Based on these findings, we propose a possible working model: changes in lipid composition and related transport processes may have created a muscle lipid environment potentially conducive to carotenoid distribution or retention, ultimately leading to deeper yellow coloration in common carp muscle (Figure 10). The results of this study provide in-sights into the formation of fish muscle color and lay the foundation for guiding the breeding of yellow-fleshed common carp strains.

Several limitations should be considered when interpreting these findings. Because the fish were wild-caught, previous dietary history and variation in age, sex, maturity stage, genetic background, and physiological status could not be fully controlled. Individual carotenoids were not absolutely quantified, and their specific contributions to muscle coloration therefore remain unresolved. In addition, carotenoid localization and retention within muscle tissue were not directly evaluated. Future studies using controlled feeding and breeding experiments, targeted quantitative carotenoid analysis, histological or imaging-based localization, direct assessment of carotenoid retention, and larger independent validation cohorts are needed to test the proposed mechanism.

## Figures and Tables

**Table 1:** Fatty acid composition and crude fat content in YC and NC muscle.

Content	YC	NC	*p* Value
C14:0	4.47 ± 0.45 a	1.15 ± 0.14 b	<0.001
C16:0	26.02 ± 0.20 a	17.87 ± 0.67 b	<0.001
C18:0	8.49 ± 0.45 a	4.52 ± 0.16 b	<0.001
C20:0	3.78 ± 0.43 a	0.61 ± 0.07 b	<0.001
SFA	42.76 ± 1.34 a	24.15 ± 0.99 b	<0.001
C14:1n-5	1.15 ± 0.12 a	0.32 ± 0.04 b	<0.001
C16:1n-7	8.76 ± 0.39 a	2.33 ± 0.13 b	<0.001
C18:1n-9	23.25 ± 3.58 b	33.62 ± 1.52 a	<0.001
C20:1n-9	3.30 ± 0.51 a	2.11 ± 0.08 b	<0.001
MUFA	36.46 ± 3.24	38.37 ± 1.59	0.197
C18:2n-6	5.90 ± 2.35 b	33.26 ± 0.37 a	<0.001
C18:3n-6	1.67 ± 0.13 a	1.01 ± 0.11 b	<0.001
C20:4n-6	3.60 ± 0.25 a	2.24 ± 0.41 b	<0.001
C22:2n-6	0.36 ± 0.02 a	0.08 ± 0.02 b	<0.001
n-6 PUFA	11.53 ± 2.46 b	36.60 ± 0.55 a	<0.001
C18:3n-3	1.10 ± 0.19 a	0.05 ± 0.02 b	<0.001
C20:5n-3	3.91 ± 0.30 a	0.13 ± 0.03 b	<0.001
C22:6n-3	4.24 ± 0.44 a	0.72 ± 0.08 b	<0.001
n-3 PUFA	9.25 ± 0.64 a	0.89 ± 0.12 b	<0.001
PUFA	20.77 ± 2.37 b	37.49 ± 0.66 a	<0.001
n-3/n-6	0.83 ± 0.17 a	0.02 ± 0.00 b	<0.001
Crude fat	27 ± 5	26 ± 4	0.737

Note: Fatty acid values are expressed as percentages of total identified fatty acids, whereas crude fat content is expressed as a percentage of muscle dry weight (DW). Data are presented as mean ± SD (*n* = 7 per group). Different lowercase letters within the same row indicate a significant difference between Yellow muscle common carp (YC) and Normal muscle common carp (NC) (*p* < 0.05). SFA, saturated fatty acids; MUFA, monounsaturated fatty acids; PUFA, polyunsaturated fatty acids; n-3 PUFA, n-3 polyunsaturated fatty acids; n-6 PUFA, n-6 polyunsaturated fatty acids; EPA, eicosapentaenoic acid; DHA, docosahexaenoic acid.

## Data Availability

The raw sequencing data reported in this paper will be released in NCBI (https://www.ncbi.nlm.nih.gov/ (accessed on 7 September 2026)) under BioProject accession number PRJNA1525809.

## Supplementary Materials

The following supporting information can be downloaded at: https://www.mdpi.com/article/10.3390/ani16213331/s1, Figure S1: Multi-omics mapping of DEGs, DAPs, and DAMs onto key lipid metabolic pathways; Table S1: Differentially abundant carotenoid-related metabolites detected in muscle; Table S2: Primer sequences used for quantitative real-time PCR.

## Abbreviations

The following abbreviations are used in this manuscript:

YC	yellow muscle common carp
NC	normal muscle common carp
CIELAB	CIE 1976 L*a*b* color space
L*	lightness
a*	redness
b*	yellowness
qRT-PCR	quantitative real-time PCR
PUFA	polyunsaturated fatty acids
MUFA	monounsaturated fatty acids
SFA	saturated fatty acids
EPA	eicosapentaenoic acid
AA	arachidonic acid
DHA	docosahexaenoic acid
DEGs	differentially expressed genes
DAPs	differentially abundant proteins
DAMs	differentially abundant metabolites
