# Supplementary material for: Characterising the Yellow Muscle Phenotype of Common Carp by Muscle Carotenoids, Fatty Acid Composition and Multi-Omics Analysis
Source: Animals (Basel). 2026 Oct 22;16(21):3331. doi: 10.3390/ani16213331 (PMC13761156; doi:10.3390/ani16213331)
Supplement: Supplementary file 1 [file animals-16-03331-s001.zip › Supplementary Tables.pdf]

Table S1. Differentially abundant carotenoid-related metabolites detected in muscle.

**Metabolite**	**FC**	**VIP**	***P* value**	**FDR**
Canthaxanthin	1.16	1.59	8.05E-05	0.00063
α-Cryptoxanthin	1.08	1.10	0.004	0.0190

Table S2. Primer sequences used for quantitative real-time PCR.

**Gene**	**Sequence (5′–3′)**	**Accession number**
gapdh	F: TCCGTCTTGAGAAACCTGCC<br>R: GTGGATACCACCTGGTCCTCT	XM_042740752.1
apol1	F: AGAAGATGAGGAGGATCTTGATGGC<br>R: AAGCCTTTGCTTCCTCTTTCTCTGT	XM_042718661.1
apobec2a	F: TCATCACCGGGGATCGCAT<br>R: GGTCCACCAGGTAACACAAGAA	XM_042711610.1
fads2	F: AGGTTCGGCACATACACCTG<br>R: GGGTGTCTCTTCACCCACTG	XM_042753409.1
cd36	F: TGGACGTCGGTAGACATTGC<br>R: TGTACGGTCCTTTCTGCACC	XM_042742724.1
cpt1b	F: CATGACTGGAGCTGGGATCG<br>R: CTGTTTGAGGAAGGGCGAGT	XM_042744408.1
lpl	F: AGTTAAGGTCCATTTCTTCGGCA<br>R: TTCAAAGCAGGCAAAATGTAGGG	XM_019064371.2
fabp3	F: TGAAACATTCGTGGGAACATGGA<br>R: CGAAACCCACACCTAGAGCCT	XM_019119618.2
bco2	F: TCATGCGCAGAATTGACCCT<br>R: TGCTCCGTTGCGGTCATAAT	XM_042754413.1
scarb1	F: TCATCGATATCCACCCGGAGAC<br>R: CTCTTCAAACCAGATCATGGGC	XM_042733125.1
rpl8	F: TCCGTGGTGTGGCTATGAAC<br>R: CCTGGACGGTCTTTGTTCCA	XM_042731504.1
